# Supplementary material for: Explaining the power-law distribution of human mobility through transportation modality decomposition
Source: Sci Rep. 2015 Mar 16;5:9136. doi: 10.1038/srep09136 (PMC5375979; doi:10.1038/srep09136)
Supplement: Supplementary Information [file srep09136-s1.pdf]

Supplementary Information for Explaining the Power-law Distribution of Human Mobility Through Transportation Modality Decomposition

Kai Zhao, Mirco Musolesi, Pan Hui, Weixiong Rao, Sasu Tarkoma

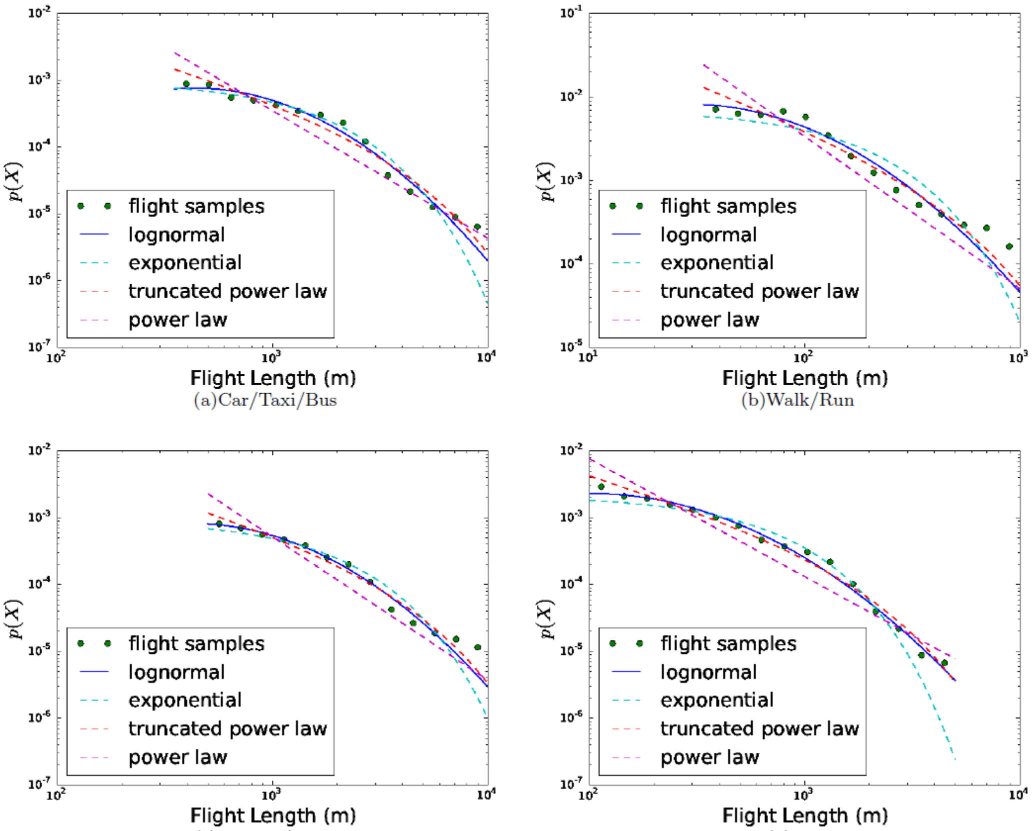

(a)Car/Taxi/Bus (b)Walk/Run (c)Subway/Train (d)Bike

Supplementary Figure S1. Lognormal fit for single transportation mode in the Nokia MDC dataset. (a-d) Flight distribution of all transportation modes (Car/Taxi/Bus, Walk/Run, Subway/Train, Bike). The green points refer to the flight length samples obtained from the Nokia MDC dataset, while the solid blue line represents the best fitted distribution according to Akaike weights. The flight length distribution in each transportation mode is well fitted with a lognormal distribution.

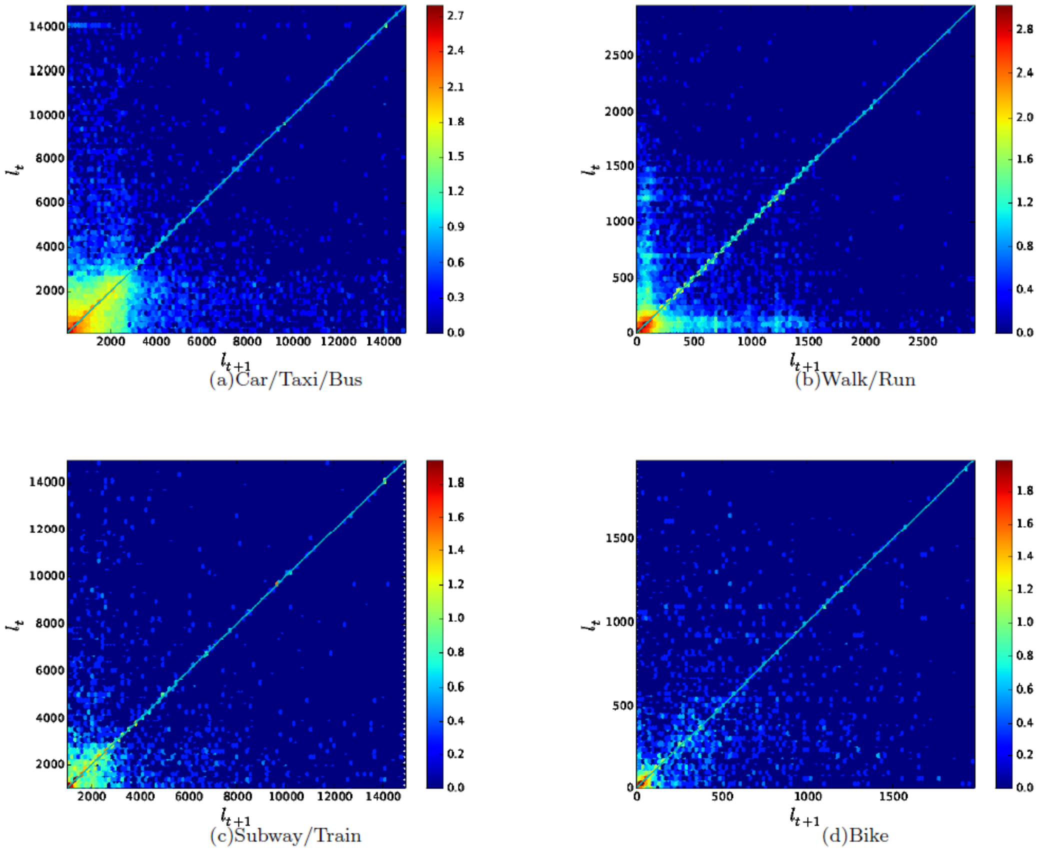

Supplementary Figure S2. Flight length correlation for each transportation mode. (a-d) Consecutive Flight length correlation of all transportation modes (Car/Taxi/Bus, Walk/Run, Subway/Train, Bike) in the Nokia MDC dataset. A high density of points are near diagonal line $l_t = l_{t+1}$, identifying a small difference $l_{t+1} - l_t$ in the same transportation mode between two time steps.

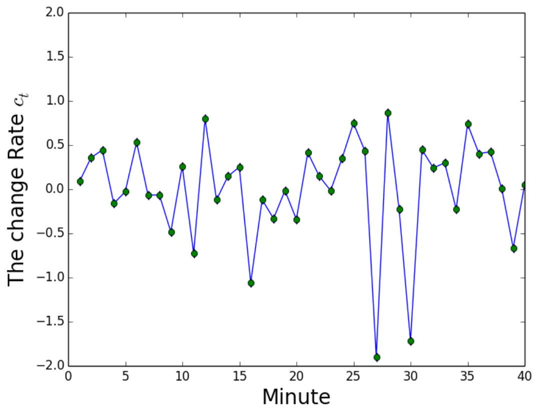

Supplementary Figure S3. The change rate of the Car/Taxi/Bus mode in the Geolife dataset. The change rate is defined as the relative change of length between two consecutive flights with the same transport mode. From the figure we observe that the change rate are uncorrelated from one time interval to the other.

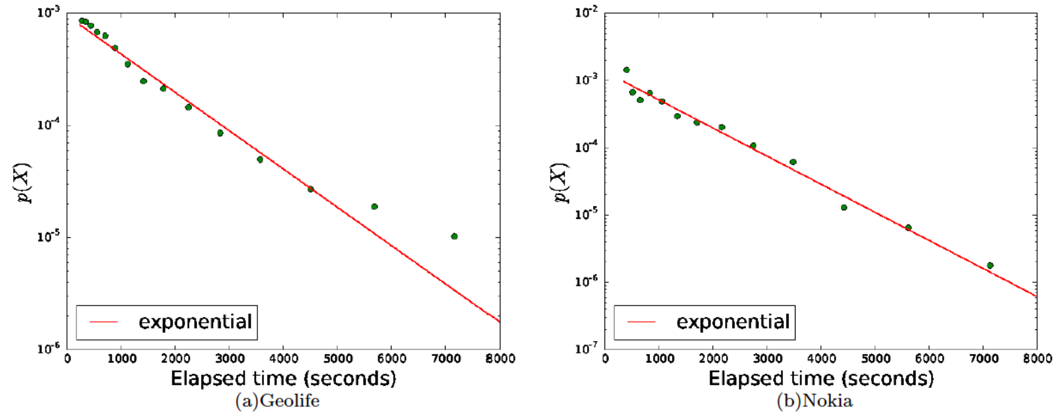

Supplementary Figure S4. Exponential elapsed time. The elapsed time t is weighted exponentially between the different transportation modes. The exponentially weighted time interval is mainly due to a large portion of Walk/Run flight intervals.

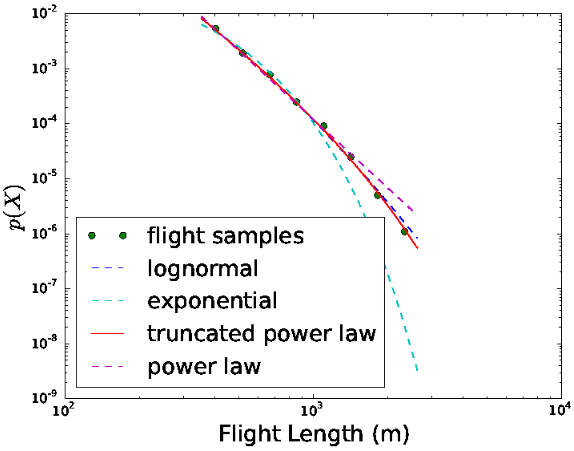

Supplementary Figure S5. The power-law distribution of street length. This figure shows that the fitted road length distribution is very different to our truncated power-law fit in flights distribution. The flight length tails in the human mobility is much larger than those shown in this figure.

Distribution	Probability density function (pdf)
Truncated power-law	$Cx^{-\alpha}e^{-\gamma x}$
Lognormal	$\frac{1}{x\sigma\sqrt{2\pi}}exp[-\frac{(ln(x)-\mu)^2}{2\sigma^2}]$
Power-law	$Cx^{-\alpha}$
Exponential	$\lambda e^{-\lambda x}$

Supplementary Table S1. Fitted distributions.

Geolife	Truncated Power-law	Lognormal	Power-law	Exponential
Overall	1.0000	0.0000	0.0000	0.0000
Car/Bus/Taxi	0.0000	1.0000	0.0000	0.0000
Subway/Train	0.0000	1.0000	0.0000	0.0000
Walk/Run	0.0000	1.0000	0.0000	0.0000
Bike	0.0000	1.0000	0.0000	0.0000
**Nokia MDC**	**Truncated Power-law**	**Lognormal**	**Power-law**	**Exponential**
Overall	1.0000	0.0000	0.0000	0.0000
Car/Bus/Taxi	0.0000	1.0000	0.0000	0.0000
Subway/Train	0.0000	1.0000	0.0000	0.0000
Walk/Run	0.0000	1.0000	0.0000	0.0000
Bike	0.0000	1.0000	0.0000	0.0000

Supplementary Table S2. Akaike weights of fitted distributions in the Geolife and the Nokia MDC datasets.

Geolife	Pearson correlation r	p
Car/Bus/Taxi	0.3640	0.0000
Subway/Train	0.6445	0.0000
Walk/Run	0.5402	0.0000
Bike	0.5584	0.0000
**Nokia MDC**	**Pearson correlation r**	**p**
Car/Bus/Taxi	0.3980	0.0000
Subway/Train	0.4681	0.0000
Walk/Run	0.4570	0.0000
Bike	0.5291	0.0000

Supplementary Table S3. Pearson correlation coefficient for consecutive flights length in the Geolife and the Nokia MDC dataset.

Geolife	Pearson correlation r	p
Car/Bus/Taxi	0.0669	0.0000
Subway/Train	0.0705	0.0000
Walk/Run	0.1342	0.0000
Bike	0.1121	0.0000
Nokia MDC	Pearson correlation r	p
Car/Bus/Taxi	0.0292	0.0001
Subway/Train	0.0282	0.0373
Walk/Run	0.0596	0.0000
Bike	0.1288	0.0020

Supplementary Table S4. Pearson correlation coefficient for the change rate in the Geolife and the Nokia MDC dataset.

SUPPLEMENTARY NOTE 1

Given

$$P(x) = \int_{t=0}^{\infty} \lambda exp(-\lambda t) \frac{1}{x\sqrt{2\pi\sigma^2 t}} exp[-\frac{(\ln(x) - \mu t)^2}{2\sigma^2 t}]dt. \quad (1)$$

The calculation to obtain α' is as follows,

$$\begin{aligned}
P(x) &= \int_{t=0}^{\infty} \lambda exp(-\lambda t) \frac{1}{x\sigma\sqrt{2\pi t}} exp[-\frac{(\ln(x) - \mu t)^2}{2\sigma^2 t}]dt \\
&= \frac{\lambda}{\sigma}\frac{1}{\sqrt{2\pi}} x^{-1} \\
&\int_{t=0}^{\infty} exp(-\lambda t) exp[-\frac{(\ln(x) - \mu t)^2}{2t\sigma^2}]\frac{1}{\sqrt{t}}]dt \\
&= \frac{\lambda}{\sigma}\frac{1}{\sqrt{2\pi}} x^{-1} \\
&\int_{t=0}^{\infty} exp[\frac{-(\ln(x) - \mu t)^2 - 2\lambda\sigma^2 t}{2t\sigma^2}]\frac{1}{\sqrt{t}}]dt \\
&= \frac{\lambda}{\sigma}\frac{1}{\sqrt{2\pi}} x^{-1} exp(\frac{\ln x\mu}{\sigma^2}) \\
&\int_{t=0}^{\infty} exp[-(\frac{\mu^2 + 2\lambda\sigma^2}{2\sigma^2})t - \frac{(\ln x)^2}{2\sigma^2}\frac{1}{t}]\frac{1}{\sqrt{t}}]dt.
\end{aligned}$$

Using the substitution $t = u^2$ gives

$$\begin{aligned}
P(x) &= \frac{\lambda}{\sigma}\frac{1}{\sqrt{2\pi}} x^{-1} exp(\frac{\ln x\mu}{\sigma^2}) \\
&\int_{u=0}^{\infty} exp[-(\frac{\mu^2 + 2\lambda\sigma^2}{2\sigma^2})u^2 - \frac{(\ln x)^2}{2\sigma^2}\frac{1}{u^2}]\frac{1}{\sqrt{u^2}}]2udu.
\end{aligned}$$

Let $a = \frac{\mu^2+2\lambda\sigma^2}{2\sigma^2}$ and $b = (\ln x)^2 2\sigma^2$, from the integral table we get

$$\int_{u=0}^{\infty} exp(-au^2 - \frac{b}{u^2}) = \frac{1}{2}\sqrt{\frac{\pi}{a}} exp(-2\sqrt{ab}),$$

which helps us to get the expression for $P(x)$,

$$\begin{aligned}
P(x) &= \frac{\lambda}{\sigma\sqrt{\frac{\mu^2}{\sigma^2} - 2\lambda^2}} x^{-(1-\frac{\mu}{\sigma^2}+\frac{\sqrt{\mu^2+2\lambda\sigma^2}}{\sigma^2})} \\
&= \frac{\lambda}{\sigma\sqrt{\frac{\mu^2}{\sigma^2} - 2\lambda^2}} x^{-\alpha'}.
\end{aligned}$$

The expression for α' is

$$\alpha' = 1 - \frac{\mu}{\sigma^2} + \frac{\sqrt{\mu^2 + 2\lambda\sigma^2}}{\sigma^2}.$$

Here the μ and the σ^2 are the normalized mean and variance of the change rate, while the λ is the exponential parameter of elapsed time between different transportation modes. We normalize the μ and σ^2 of different transportation modes following $\mu = (\mu_{Car/Bus/Taxi} + \mu_{Subway/Train} + \mu_{Walk/Run} + \mu_{Bike})/4$ and $\sigma^2 = (\sigma^2_{Car/Bus/Taxi} + \sigma^2_{Subway/Train} + \sigma^2_{Walk/Run} + \sigma^2_{Bike})/4$. Note here $\mu_{Car/Bus/Taxi}$, $\mu_{Subway/Train}$, $\mu_{Walk/Run}$, μ_{Bike} and $\sigma_{Car/Bus/Taxi}$, $\sigma_{Subway/Train}$, $\sigma_{Walk/Run}$, σ_{Bike} represent the mean and standard deviation of the change rate in each transportation modes in both datasets, as shown in the Table. 2. The mean value μ is 5.54 and 6.05 and the variance σ^2 is 0.5954 and 1.0165 in the Geolife dataset and in the Nokia MDC dataset respectively. Combining the fitted exponential parameter $\lambda = 3.16$ in the Geolife dataset and $\lambda = 2.53$ in the Nokia MDC dataset, we obtain the final $\alpha' = 1.55$ in the Geolife dataset, which is close to the original parameter $\alpha = 1.57$, and $\alpha' = 1.40$ in the Nokia MDC dataset, which is close to the original parameter $\alpha = 1.39$.
